# Tissues and mechanisms associated with Verticillium wilt resistance in tomato using bi-grafted near-isogenic lines

**DOI:** 10.1093/jxb/erad182

**Published:** 2023-05-15

**Authors:** Yeonyee Oh, Thomas Ingram, Reza Shekasteband, Tika Adhikari, Frank J Louws, Ralph A Dean

**Affiliations:** Department of Entomology and Plant Pathology, North Carolina State University, Raleigh, NC 27695, USA; Department of Entomology and Plant Pathology, North Carolina State University, Raleigh, NC 27695, USA; Department of Horticultural Science, North Carolina State University, Raleigh, NC 27695, USA; Department of Entomology and Plant Pathology, North Carolina State University, Raleigh, NC 27695, USA; Department of Entomology and Plant Pathology, North Carolina State University, Raleigh, NC 27695, USA; Department of Horticultural Science, North Carolina State University, Raleigh, NC 27695, USA; Department of Entomology and Plant Pathology, North Carolina State University, Raleigh, NC 27695, USA; University of Ghent, Belgium

**Keywords:** Defense response, gene expression, grafting, secreted proteins, tomato, *Verticillium dahliae*

## Abstract

Host resistance is the primary means to control *Verticillium dahliae*, a soil-borne pathogen causing major losses on a broad range of plants, including tomato. The tissues and mechanisms responsible for resistance remain obscure. In the field, resistant tomato used as rootstocks does not confer resistance. Here, we created bi-grafted plants with near-isogenic lines (NILs) exhibiting (*Ve1*) or lacking (*ve1*) resistance to *V. dahliae* race 1. Ten days after inoculation, scion and rootstock tissues were subjected to differential gene expression and co-expression network analyses. Symptoms only developed in susceptible scions regardless of the rootstock. Infection caused more dramatic alteration of tomato gene expression in susceptible compared with resistant tissues, including pathogen receptor, signaling pathway, pathogenesis-related protein, and cell wall modification genes. Differences were observed between scions and rootstocks, primarily related to physiological processes in these tissues. Gene expression in scions was influenced by the rootstock genotype. A few genes were associated with the *Ve1* genotype, which was independent of infection or tissue type. Several were physically clustered, some near the *Ve1* locus on chromosome 9. Transcripts mapped to *V. dahlia*e were dominated by secreted candidate effector proteins. These findings advance knowledge of molecular mechanisms underlying the tomato–*V. dahliae* interaction.

## Teaser

Using grafted tomato plants, the tissues and mechanisms of host resistance to Verticillium wilt, a destructive soil-borne pathogen, were examined. Foliar tissues determined resistance whereas susceptible tissues responded most dramatically to infection.

## Introduction

Root-infecting pathogens represent a major challenge both to agricultural production and to elucidating the underlying molecular mechanisms of pathogenesis and host defense. Pathogens such as *Verticillium dahliae* that then invade the xylem, the major water- and mineral-conducting system of plants, and cause vascular wilts are particularly destructive. *Verticillium dahliae* is a pervasive soil-borne pathogen that causes Verticillium wilt on several hundred plant species, including tomato and many other important agronomic crops (Fradin and Thomma, 2006; Acharya *et al.*, 2020). The fungus persists in the soil as hardy microsclerotia for many decades that are near impossible to eliminate by chemical treatments. In the presence of root exudates, microsclerotia germinate and the fungus enters nearby lateral roots. Within a few days, the fungus migrates to the xylem tissues, where conidia are produced and carried into the above-ground parts even in resistant plants (Sewell and Wilson, 1964; Garas *et al.*, 1986). Subsequently, in susceptible plants, conidia germinate and the pathogen continues to proliferate in the vascular system and subsequently into adjacent tissues (Gold and Robb, 1995; Heinz *et al.*, 1998; Chen *et al.*, 2004). Leaf tips eventually turn yellow, particularly on the lower leaves where V-shaped necrotic lesions occur. Tomato plants become stunted, with extensive defoliation that under severe conditions leads to plant death. A primary strategy to manage wilts and other soil-borne pathogens including *V. dahliae* is through host resistance.

The presence of soil, complexity, and confounding effects of other rhizosphere microbiota, and the need to use invasive or destructive measures to access infected tissues make investigations of root-infecting pathogens difficult. Consequently, the bulk of research has focused on foliar pathogens that cause local lesions and invoke typical plant defense responses involving reactive oxygen species (ROS), the release of Ca^2+^, and rapid host cell death known as the hypersensitive response (HR) (Heinze and Andrus, 1945; Levine *et al.*, 1994; Lamb and Dixon, 1997; Morel and Dangl, 1997). These studies led to the concept of two layers of plant defense first proposed in the zig–zag model of Jones and Dangl (2006). The first layer, considered as the basal defense, is referred to as pattern triggered immunity (PTI) and involves surface pattern recognition receptors (PRRs) recognizing conserved features of microorganisms and pathogens (DeFalco and Zipfel, 2021). The model offers that a second layer evolved to combat adapted pathogens, referred to as effector triggered immunity (ETI), and involves cytoplasmic events typically mediated by nucleotide-binding leucine-rich (NB) proteins that recognize specific molecules (effectors) from a particular race of a pathogen (Cui *et al.*, 2015). While once thought as discrete, the mechanistic boundaries between PTI and ETI have blurred over the past decade or so, with numerous exceptions to the model, in particular the characterization of apoplastic effectors being detected by receptor-like proteins/kinases (RLP/RLKs) similar to PRRs (Dodds and Rathjen, 2010; Cook *et al.*, 2015). Indeed, recent evidence indicates that much of the activated defense machinery assigned to these pathways is common, highly interconnected, and mutually dependent (Kadota *et al.*, 2019; Ngou *et al.*, 2021; Yuan *et al.*, 2021). However, studies of resistance mechanisms to xylem-infecting pathogens are more limited, and whether similar defense pathways, particularly the HR, occur in response to these adapted pathogens requires further investigation (De Coninck *et al.*, 2015; Chuberre *et al.*, 2018; Ding *et al.*, 2021; Kashyap *et al.*, 2021).

Race-specific resistance to root-infecting pathogens, aligning with the gene-for-gene concept, has been characterized in several instances and shown to be mediated by single genes (Kashyap *et al.*, 2021). A number of the resistance gene products are membrane receptors. In tomato, examples include I, an RLP (perception of Avr1), and I3, an S-RLK (perception of Avr3) conferring resistance to *Fusarium oxysporum* f. sp. *lycopersici* (*Fol*) (Catanzariti *et al.*, 2015, 2017). *Ve1*, which mediates resistance to race 1 *V. dahliae* by perception of VdAve1, also encodes a membrane receptor (Kawchuk *et al.*, 2001). Wilt resistance, however, is typically multilayered, involving different tissues and cell layers, underscoring observations that resistance often is polygenic. In many instances, resistance to vascular pathogens, including *V. dahliae*, is not absolute and involves considerable, but reduced, growth (or reproduction) of the pathogen in the resistant tissues. *Verticillium dahliae* is primarily restricted to xylem tissues, and pathogen levels fluctuate in resistant tomato plants undergoing rounds of suppression presumably succumbing to host defenses (Chen *et al.*, 2004). Co-expression of Ve1 and VdAve1 in *Nicotiana tabacum* foliar tissues evoked an HR-like response (De Jonge *et al.*, 2012), although evidence for HR in wild-type resistant plants is lacking. It has long been suggested that disease resistance largely depends on the speed of the defense response, suggesting that resistance is based on a quantitative response rather than qualitative differences (Navarro *et al.*, 2004; van Esse *et al.*, 2009). Resistance to wilt pathogens, including *V. dahliae*, has been reported to involve the accumulation of phenolics, and formation of physical barriers, such as tyloses and gels, suberin (aromatics conjugated with aliphatic compounds), and callose and lignin deposition in xylem walls to contain the pathogen. However, similar responses have been also reported during compatible interactions (De Coninck *et al.*, 2015; Kashyap *et al.*, 2021). Other studies have observed differences in the patterns of gene expression between an incompatible and compatible interaction, suggesting qualitative differences (Xu *et al.*, 2011; Manzo *et al.*, 2016; Chang *et al.*, 2021). However, defense gene expression typically differs in response to root-/xylem-invading pathogens compared with foliar pathogens (Zhang, 1998; Fradin *et al.*, 2009, 2011; Robb *et al.*, 2009; van Esse *et al.*, 2009; De Coninck *et al.*, 2015). Thus, the defense mechanisms related to *Ve1*-mediated resistance to *V. dahliae* in tomato tissues remain enigmatic.

Grafted plants have been used extensively to improve yield and disease resistance in many high-value crops, including tomato (Thies, 2021). Indeed, the use of rootstocks with resistance to race 1 or 2 of Fusarium wilt, caused by *Fol*, as well as bacterial wilt, caused by *Ralstonia solanacearum*, is generally an effective management practice (Rivard and Louws, 2008; Louws *et al.*, 2010; Thies, 2021). On the other hand, the role the rootstock plays in resistance to *V. dahliae* is less clear-cut (Louws *et al.*, 2010; Acharya *et al.*, 2020). Work using tomato Craigella (*ve1*) and a near-isogenic line (NIL) containing the *Ve1* gene has shown that *V. dahliae* is fully systemic in both the resistant (*Ve1*) and susceptible (*ve1*) plants within 2–3 d, suggesting that the roots do little to restrict entry into the xylem and subsequent upward movement into the foliar tissues (Chen *et al.*, 2004). These NILs have been used previously in grafting experiments to examine the basis of resistance, including limited microarray and proteomics analyses (Robb *et al.*, 2009, 2012).

In this study, to obtain deep and comprehensive insight into the tissue basis and mechanisms of host resistance to *V. dahliae* race 1, we evaluated differential gene expression and co-expression network analyses using next-generation whole-genome expression data from scions and rootstocks of grafted plants 10 days after inoculation (DAI) or mock inoculation. We hypothesized that at 10 DAI, defense responses would be activated in resistant tissues. Our hypothesis was supported by two previous observations. (i) Tissue sampling and PCR analysis showed that the fungus was established in rootstock and scion tissues of both susceptible and resistant plants within a few days of inoculation. (ii) In our hands, symptoms (wilting) in susceptible plants began to be evident 2 weeks after inoculation on grafted plants. For these experiments, we used bi-branched plants, where scions from the Craigella NILs of tomato containing (*Ve1*) or lacking resistance (*ve1*) to race 1 of *V. dahliae* were both grafted onto rootstocks of either the susceptible or resistant genotypes. Our findings advance new fundamental insights into tissue- and genotypic-specific responses to *V. dahliae* infection and, as a sidebar, our findings highlight the benefits and limitations of using NILs.

## Materials and methods

### Establishment of bi-branched and cross-grafted tomato plants and fungal inoculation

**Fig. 1.**
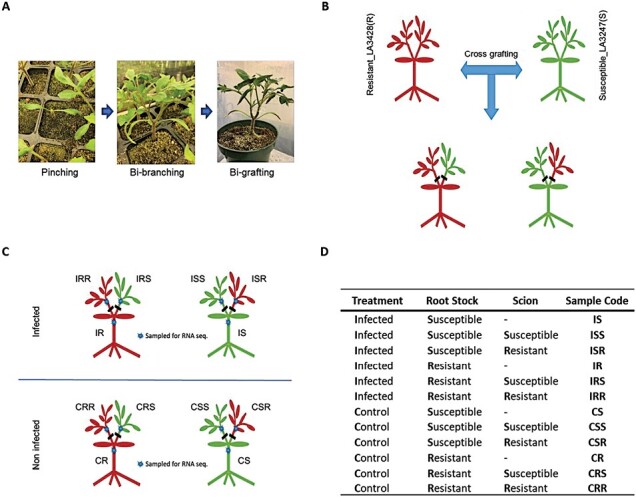
Generation of bi-grafted tomato plants and sample collection for R seq. The near-isogenic lines LA3247 (*ve1*) and LA3428 (*Ve1*) were trained to be bi- branched (panel **A**) and then cross-grafted (panel **B**). The resistant tomato line (R, LA3428) is depicted in red, and the susceptible line (S, LA3247) is in green. Samples are coded based on the treatment (infected (I) and mock-infected control (C), the rootstock genotype (S, R) and the scion genotype (S, R) (panels **C** and **D**). For example, ISR = resistant scion tissue from infected plant with susceptible rootstock.

Susceptible tomato line Craigella (LA3247, *ve1*) and a near-isogenic resistant line (LA3428, *Ve1*) were obtained from the Tomato Genetics Cooperative, Davis, CA, USA. Plants were grown in a soil-less mix (SunGro Professional Growing Mix) and placed in a growth room at 24 °C/22 °C with a 14 h/10 h day/night period (Gold and Robb, 1995). Two-week-old tomato plants were trained to be bi-branched by pruning. Two weeks later, bi-grafted tomato plants were generated, on which susceptible and resistant scions were grafted to each branch of either susceptible or resistant rootstock (Fig. 1). The grafted plants were kept in a humid chamber for 10 d for healing.

### Fungal inoculation, plant sample collection, and RNA and DNA extraction

*Verticillium dahliae* isolate Le1087 was collected from tomato in California in the 1950s and is considered a race 1 reference isolate (Short *et al.*, 2014). Based on pathogenicity tests on the tomato NILs and the use of a gene knockout of VdAve1 in Le1087, Le1087 was confirmed as race 1 and that resistance depends on the co-existence of tomato *Ve1* and fungal VdAve1. *Verticillium dahliae* race 1, Le1087 was grown on PDA agar media at 28 °C in the dark for 7 d. The fungal spores were collected with distilled water and the concentration adjusted to 2 × 10^7^ spores ml^–1^. Plants were uprooted, and the roots were rinsed with running water, dipped into the spore solution for 15 min, and replanted. For the non-infected control treatment, plants were dipped into distilled water.

**Fig. 2.**
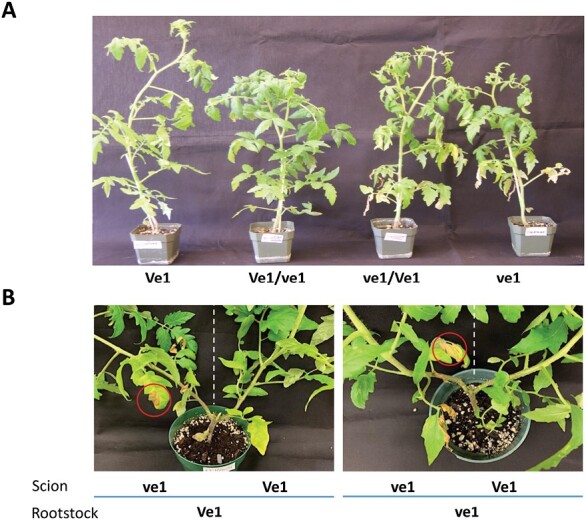
Verticillium wilt disease progress in grafted plants. (**A)** Tomato plants were grafted reciprocally using LA3428 (Ve1) and LA3247 (ve1), and infected with the race 1 strain Le1087. Wilting was evident on the non-grafted Ve1- plant (far right) and grafted plant with ve1 scion over the Ve1 rootstock (ve1/Ve1, second from right). (**B**) Ve1 and ve1 rootstocks were bi-grafted with both Ve1 (right of white dashed line) and ve1 (left of white dashed line) scions. Typical V-shaped necrotic lesions (circled in red) were evident on the ve1 scions on both types of rootstock. Photographs were taken 4 weeks after inoculation.

At 10 DAI, 24 plants with ~1 cm stems (2 rootstocks×2 scions×2 treatments×3 replicates) were collected at the branching point of the first leaf on each scion, and 12 hypocotyl stem segments of ~1 cm from rootstocks (2 rootstock×2 treatment×3 replicates) were collected from just under the cotyledon (Fig. 2). Tissue samples were kept at –80 °C before further processing. Total RNAs were prepared from the 36 plant samples using the RNAeasy Plant Mini Kit (Qiagen Inc.) and the RNA quality was checked by Bioanalyzer. Total DNA was isolated using the DNeasy Plant Mini Kit (Qiagen Inc).

### Fungal biomass measurement by quantitative real-time PCR

The degree of fungal colonization was determined by the amplicon levels of *V. dahliae* using the DF-DR primers (Pérez-Artés *et al.*, 2005) compared with the amplification of the tomato EXP (SOLYC07G025390) gene in 100 ng of total genomic DNA from each sample. The relative amplicon abundance was calculated using the 2^−ΔΔCt^ method (Livak and Schmittgen, 2001) from two biological and two technical replicates.

### Next-generation RNA sequencing and data analysis

Total RNA samples were submitted to the Genomic Sciences Laboratory, North Carolina State University, Raleigh, NC, USA. The cDNA libraries were constructed using the NEBNext® Ultra™ Directional RNA Library Prep Kit for Illumina (New England BioLabs, Ipswich, MA, USA). An Illumina NovaSeq 6000 instrument was used to generate RNA-seq data in 150 bp paired-end mode. The sequencing data were processed using the Galaxy platform (https://usegalaxy.org/). The quality of the raw data was checked through FastQC (Andrews, 2010) and the low-quality reads and adapter sequences were eliminated using the Trim Galore function.

For tomato gene expression, reads were mapped against the Heinz 1706 genome assembly SL4.0 using Hisat2 version 2.1.0 (Kim *et al.*, 2019). The htseq-count function was used to calculate the number of reads mapping to each feature in the annotated gene model (ITAG4.0 version) (Anders *et al.*, 2015). Using a web-based application, iDEP (Ge *et al.*, 2019), genes with >0.5 counts per million (CPM) in at least one library sample were considered to be expressed, and the expression profile of 22 312 genes from the SL4.0 genome was obtained.

Raw counts data were normalized to CPM and transformed to log2(CPM+1) using the R package EdgeR (Robinson *et al.*, 2010). Hierarchical clustering and principal component analysis (PCA) were performed using the default setting in iDEP. Differentially expressed genes (DEGs) were identified by applying a minimum 2-fold change between comparisons with a <10% false discovery rate (FDR).

For the study of *V. dahliae* gene expression, reads were mapped to two publicly available annotated *V. dahliae* reference genomes, JR2 (BioProject Accession PRJNA175765) and VdLs17 (BioProject PRJNA28529), from the NCBI. JR2 was isolated from tomato and was predicted to have 11 426 genes including race 1 and race 2 effectors, VdAve1 and Av2 (Kadota *et al.*, 2019; Chavarro-Carrero *et al.*, 2021). Non-race 1 VdLs17 was isolated from lettuce with 10 535 predicted genes (Klosterman *et al.*, 2011).

### Gene function and network analysis

Tomato gene function was assigned based on the annotated gene model, ITAG4.0. (https://solgenomics.net/). Using the publicly available SL4.0 Gene Ontology (GO) annotation database (Bizouerne *et al.*, 2021b), GO enrichment analyses were conducted through the topGO package (v2.34.0) in R (Alexa and Rahnenfuhrer, 2010) applying a Fisher’s exact test using the weight01 method. Enrichments were considered significant for GO terms with *P*-values <0.001 (Bizouerne *et al.*, 2021a).

Weighted Gene Correlation Network Analysis (WGCNA, v1.68) in the R package was used to find clusters (Modules) of highly correlated genes, identify highly connected (hub) genes within modules, relate modules to one another and external traits [genotype, Verticillium infection, tissue type (rootstock, scions)], and to analyze module eigengene vectors within trait modules (Langfelder and Horvath, 2008). Within WGCNA, the automatic one-step network construction was used for module detection, with power (soft threshold) set to 12, TOMType to signed, minModuleSize to 30, and mergeCutHeight to 0.35 selected. Modules significantly associated with fungal infection, tomato genotype, and tissue type were identified using a Pearson correlation between eigengene expression profiles with those traits. The hub genes were identified based on the module membership (MM; Pearson correlation between the module eigengene and the gene expression profile) and the number of genes highly connected in the module. Data were further processed and visualized using Cytoscape (v3.7.1) (Otasek *et al.*, 2019).

The Pathogen Receptor Genes (PRGs) database (http://prgdb.org/prgd/), which contains 1667 genes mapped to the SL4 tomato genome, was used to identify expressed genes annotated with a role in pathogen detection. For *V. dahliae* gene expression analyses, GO was assigned using the DAVID bioinformatics database (https://david.ncifcrf.gov/home.jsp). Signal peptides were assigned using SignalP (Almagro Armenteros *et al.*, 2019).

### Genome-wide polymorphisms

Genetic variants were called using BCFtools/1.9 (Li *et al.*, 2009) and in-house shell scripts. After variant calling, single nucleotide polymorphisms (SNPs) with a minimum mapping quality of 10, and minimum coverage of four reads at every position in the reference genome were kept to compare the identified SNPs for introgression identification. Variants from all three replicates for susceptible and resistant non-infected rootstock samples were merged, saving only monomorphic SNP sites that were present consistently in all three replicates. The two datasets were merged by TASSEL 5 (Bradbury *et al.*, 2007) to identify SNPs polymorphic between the two NILs.

## Results

### Disease development in bi-grafted tomato plants

To evaluate the efficacy of grafting to prevent disease progress, we first evaluated reciprocally grafted Verticillium wilt resistant and susceptible Craigella near-isogenic plants following inoculation with *V. dahliae* Le1087, a race 1 isolate. Disease symptoms including wilting, yellowing, and necrosis developed only in the susceptible scions regardless of the rootstock genotype for resistance. No symptoms were observed on resistant scions on susceptible rootstocks (Fig. 2A). To further interrogate the basis of resistance to *V. dahliae*, we created bi-grafted plants where rootstocks bore both a resistant and a susceptible scion, as shown in Fig. 1. Similarly, we observed wilting and necrotic symptoms only in the susceptible scions regardless of rootstock resistance in the bi-grafted plants (Fig. 2B). These findings support the importance of the scion genotype rather than the rootstock genotype for conferring *V. dahliae* race 1 resistance in tomato. At the time of tissue sampling, 10 DAI, no symptoms or significant differences in plant heights between any of the different bi-grafted plant treatments were observed (Supplementary Fig. S1).

### Summary of RNA-seq library and mapping

RNA from 36 samples [12 experimental combinations (8 from scions, 4 from rootstocks)×3 replicates as shown in Fig. 1D] yielded between 53.5 million and 93.8 million paired-end reads, with >99% being quality reads (Supplementary Table S1). Overall, 85–88% of reads mapped to the tomato reference genome. On the other hand, very few reads (<0.02% of total reads) mapped to *V. dahliae*, with a maximum of 15 831 reads in the rootstock of the susceptible line (IS). Fewer than 50 reads mapped to *V. dahliae* from non-infected tissue. The low numbers of reads mapping to *V. dahliae* precluded in-depth analysis; however, basic *in silico* functional analyses are presented, following interrogation of tomato gene expression patterns.

### Hierarchical clustering and PCA analysis of tomato gene expression

**Fig. 3.**
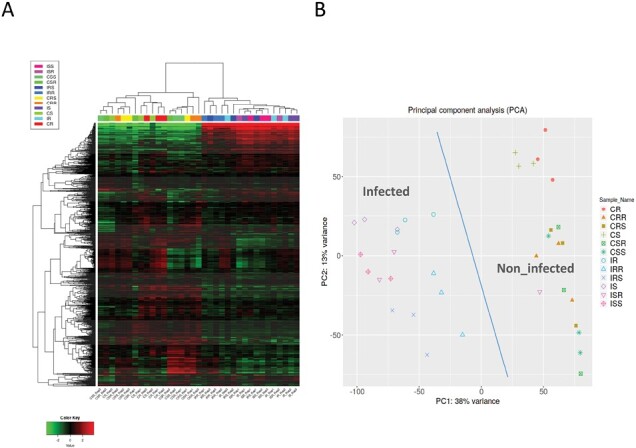
Hierarchical (panel **A**) and principal component analysis (PCA) (panel **B**) analyses of tomato gene expression patterns. See Fig. 1 for sample codes. Note: Sample ISR_Rep3 was discarded as it clustered with the non-infected control subgroup (likely due to an error during sample collection).

Hierarchical clustering revealed that infected and non-infected control samples were separated into two subgroups (Fig. 3A). Based on tissue type or genotype, the clustering of replicates for these traits within each major subgroup was not exact. However, two of the three replicates per sample typically clustered together, and rootstock and scion samples typically clustered separately.

PCA showed that overall, infected, and non-infected control samples (ISR-Rep3 excluded) clustered separately (Fig. 3B). Within non-infected samples, gene expression of rootstock and scions was distinct regardless of tomato genotype. There was no apparent difference in expression patterns between susceptible and resistant rootstocks in non-infected samples. Similarly, there was no clear separation among scion samples regardless of the type of rootstocks for non-infected plants.

Among the infected samples, there were some differences in gene expression patterns between scion and rootstock samples. Within the rootstocks, similar to non-infected samples, there was no strong partition of gene expression patterns based on genotype. Expression in scion samples was affected by the genotype of rootstocks. On susceptible rootstocks, no separation was observed between susceptible and resistant scions. In contrast, on the resistant rootstock, gene expression was different between susceptible and resistant scions.

In sum, PCA revealed a clear distinction in gene expression patterns in infected versus non-infected tissues, and some evidence for differences in gene expression patterns based on tissue (scions versus rootstocks). On the other hand, there was no obvious distinction in gene expression following infection of resistant versus susceptible tissues, although infection of resistant rootstocks appeared to influence gene expression in resistant versus susceptible scions.

### Effect of rootstock on scion gene expression

**Fig. 4.**
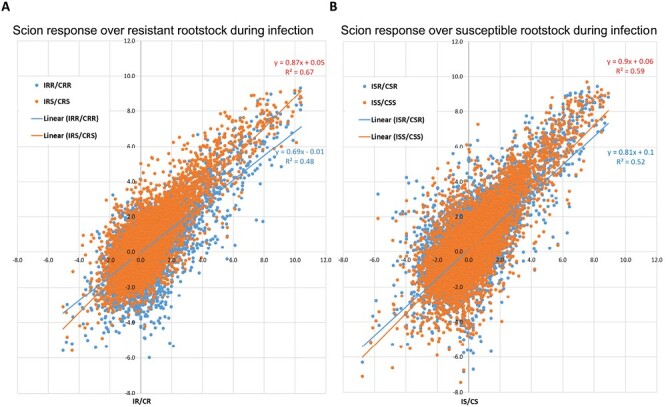
Infection gene expression response in the susceptible and resistance scions grafted to resistant and susceptible rootstocks, respectively. (A) Differential gene expression between the infected and non-infected control resistant rootstock shown as log2 fold change, (IR/CR) and was plotted against the differential gene expression in susceptible (IRS/CRS) and resistant scion (IRR/CRR). (B) Same as in (A), differential gene expression was compared between susceptible (ISS/CSS) and resistant (ISR/CSR) scions grafted to a susceptible rootstock (IS/CS).

To further investigate details of rootstock genotype effects on the infection-related gene expression in the scion, we compared expression patterns in susceptible and resistant scions. Grafted to the resistant rootstock, genes were less responsive in the resistant scion than in the susceptible scion following infection (see blue versus red regression lines, Fig. 4A). In contrast, grafted to the susceptible rootstock, infection gene responses between susceptible and resistant scions were similar (Fig. 4B). These data further clarify how the resistant rootstock influences expression in the scions evidenced by the PCA (Fig. 3B).

### Analysis of differentially expressed genes in tomato in response to *V. dahliae*

**Fig. 5.**
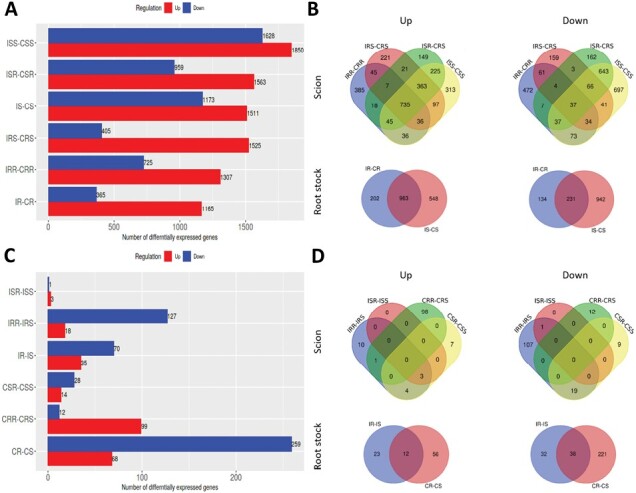
Differentially expressed genes (DEGs) in response to *V. dahliae*. (A) Pair-wise comparison between infected and non-infected tissues. See Fig. 1D for treatments. Red indicates genes up-regulated in infected tissues whereas blue indicates down-regulated genes in infected tissue. (B) Identification of common infection DEGs in scions and rootstock tissues. (C) DEGs associated with genotype by pair-wise analysis of DEGs between genotypes. Red indicates genes up-regulated in *Ve1*+ tissues whereas blue indicates genes down-regulated in *Ve1*+. (D) Identification of common DEGs between genotypes.

Infection resulted in distinct and measurable differences in gene expression in both susceptible and resistant genotypes (Fig. 5A). Based on pairwise comparisons (fold change >2 and FDR <0.1), high numbers (1538–3478) of DEGs were observed in all tissues (rootstocks and scions) following infection (IR, IS, ISS, ISR, IRS, and IRR) compared with the corresponding non-infected control, representing between 7% and 15% of all expressed genes. Overall, more DEGs were observed in susceptible (1916–3478) compared with resistant (1530–2522) tissues with more up-regulated (1165–1850) than down-regulated (365–1628) following *V. dahliae* infection.

Comparisons between infected and non-infected samples revealed 735 up-regulated DEGs common to all infected versus non-infected scion samples (out of 1307–1850 DEGs, representing >40% shared) compared with 37 down-regulated DEGs (out of 405–1628 DEGs, representing <9% shared). In rootstocks, however, 963 (56%) up- and 231 (18%) down-regulated DEGs were shared. In sum, combining data from rootstocks and scions revealed a total of 635 commonly up-regulated and 16 down-regulated DEGs (Fig. 5B).

### Differentially expressed genes associated with the *Ve1* gene in tomato

To identify DEGs associated with the *Ve1* gene, comparisons across the two genotypes were conducted. Pairwise comparisons revealed a relatively small number of DEGs, for both infected and non-infected comparisons, particularly for scions (ISR–ISS, 4 DEGs, up to 145 DEGs in IRR–IRS), although a slightly larger differential response was observed in rootstocks (IR–IS, 105 DEGs, CR–CS, 327 DEGs) (Fig. 5C). It is noteworthy that differences in gene expression were observed between the non-infected genotypes. Overall, these results suggest that although tomato responds extensively to *V. dahliae* infection, there was very little consistent difference in gene expression patterns between susceptible and resistant tissues following infection (Fig. 5D).

### Gene networking analysis

**Fig. 6.**
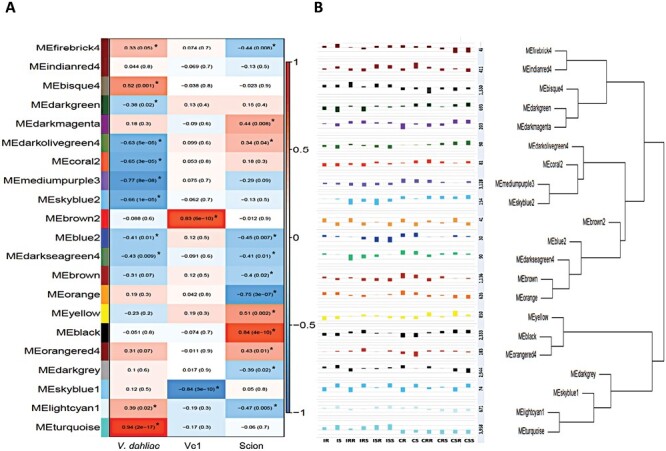
Gene expression modules. (A) The module–trait relationship. Pearson correlation between the module and traits for infection (*V. dahliae*), genotype (*Ve1*), and tissue type (scion/rootstock) were calculated. (B) Eigenvalue bar plots for the co-expression analysis. Each bar plot represents the average eigengene values across the 12 samples. The figure displays the number of genes in each of the 21 modules as well as the dendrogram showing the relationship of modules to each other. *Indicates that the module is significantly correlated to the trait. Generally, closely clustered modules showed similar connections to traits.

To gain more subtle and holistic insights into gene expression patterns based on genotype, infection, and tissue traits, we conducted co-expression network analysis employing WGCNA on the entire transcriptome dataset. Using filters and cut-offs described in the Materials and methods, 21 modules containing 22 312 genes were generated (Fig. 6). Modules ranged considerably in size; the smallest, brown2, contained 41 genes, whereas the largest, turquoise, contained 3598 genes.

#### Co-expression analyses related to infection

The module turquoise, the most positively associated with infection (*r*=0.94), contained 3958 genes and included 634 of the 635 up-regulated DEGs common across scions and rootstocks (see above). Eigenvalue bar plots for the 12 treatments revealed a consistent pattern for all infected and non-infected samples. (Fig. 6B).

Functional analysis using TopGO showed that GO categories associated with responses to various stresses including oxidative, chemical, defense responses, and biotic and external stimulation were over-represented for genes in this module. The most highly enriched GO categories included protein autophosphorylation (GO:0046777), response to chitin (GO:0010200), cellular response to abscisic acid stimulus (GO:0071215), response to salicylic acid (SA; GO:0009751), and defense response to fungus (GO:0050832). Other defense-related enriched GO terms included lignin biosynthetic process (GO:0009809) and regulation of ethylene (GO:0010104) and jasmonic acid (JA; GO:200022) signaling (Supplementary Table S2).

**Fig. 7.**
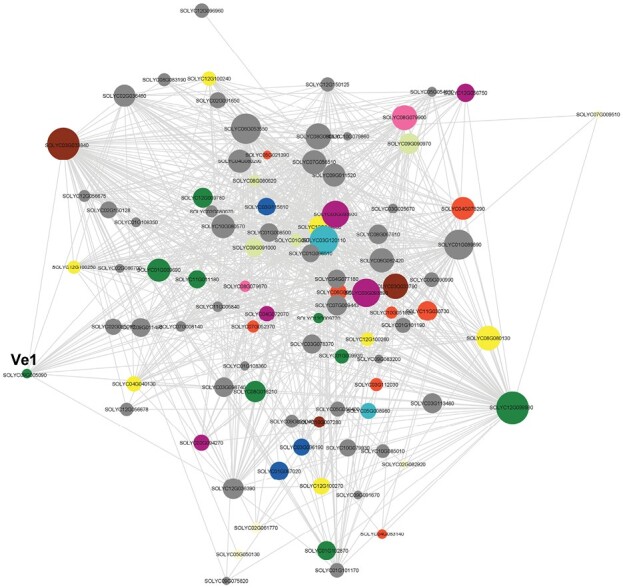
Major hub genes and their connections in the turquoise module. Major functional groups are marked in colors (ATPase_brown, TF_purple, RLP_green, RLK_blue, KIN_light blue, protease_pink, chitinase_yellow, cytochrome_orange, PR protein_light green). The size of the node represents the number of connections above the cut-off weight of 0.4, with >50 connections shown. Edges (the solid line between nodes) are shown for connection weights >0.43. AAA-ATPase At3g50940-like protein (SOLYC03G033840) and Cf2/5 homolog (SOLYC12G099980) are central hub genes.

Genes in module turquoise were the most strongly connected compared with genes in other modules; that is, they had the greatest number of genes with similar expression patterns. ‘Hub’ genes were identified, based on the number of connected genes, above a connection threshold (strength of the connections). The primary hub genes and their connections are visually represented in Fig. 7.

Primary hubs included several AAA-ATPases, pathogenesis-related proteins, receptor-like protein kinases (including *Ve1* and other Cf2/Cf5 and Cf4/Cf9 homologs), and transcription factors (see Supplementary Table S3). Other genes of note in the turquoise module included a number of key genes involved in the shikimate/lignin biosynthesis pathway including several phenylalanine ammonia-lyase genes, a cinnamyl alcohol reductase (SOLYC11G011330), and peroxidases (SOLYC05G050870 and SOLYC0G050880), as well as key genes involved in suberin formation such as several tyramine hydroxycinnamyl transferase genes and a tyramine *N*-feruloyl transferase (SOLYC08G068790).

Module mediumpurple3, containing 3128 genes, was most negatively associated (*r*= –0.77) with infection; that is, positively associated with healthy tissue (Fig. 6A). GO terms linked to development, transport, protein modification, localization, and stability were over-represented, including cellular response to freezing (GO:0071497), positive regulation of transcription by RNA polymerase II (GO:0045944), positive regulation of protein dephosphorylation (GO:0035307), histone H4 acetylation (GO:0043967), and protein K63-linked ubiquitination (GO:0070534) (Supplementary Table S2). The key hub genes in this module are listed in Supplementary Table S3.

#### Co-expression analyses related to tissue type

Several modules, in particular black (2333 genes, *r*=0.64) and orange (626 genes, *r*= –0.75), exhibited sample eigengene values consistently associated with tissue (scions versus rootstocks), (Fig. 6A, B; Supplementary Table S2). Unsurprisingly, GO terms associated with photosynthesis (GO:0009768), response to low fluence red light stimulus (GO:0010202), protein–chromophore linkage (GO:001829), regulation of auxin polar transport (GO:2000012), and cotyledon morphogenesis (GO:0048826) were over-represented in the positively associated with scion black module. In contrast, the orange module, which was negatively associated with scion (i.e. positively associated with rootstock, see Fig. 6A, B; Supplementary Table S2), contained several over-represented GO terms including chorismate (a precursor of SA). Key hub genes for this module included enzymes in the shikimate/phenylpropanoid pathway such as phenylalanine ammonia-lyase (SOLYC05G056170), 4-coumarate:CoA ligase (SOLYC03G117870), and caffeoyl-CoA *O*-methyltransferase (SOLYC02G093270) (Supplementary Table S3). These observations probably reflect the maturity of the tissues. Rootstocks are more mature than scions and therefore are expected to have greater secondary wall thickening (i.e. lignification).

#### Co-expression analyses related to genotype

Of note, we also identified two modules associated with genotype (presence of the *Ve1* gene): module brown2 containing 41 genes was positively correlated (*r*=0.83) with the presence of *Ve1*, whereas module skyblue1 containing 74 genes was negatively correlated (*r*= –0.84). Neither of these modules was correlated with infection or tissue type (Fig. 6A, B). Of the 41 genes in the brown2 module, 29 had functional annotation (Supplementary Table S4). These genes appeared to be constitutively up-regulated in resistant tissues, with several associated with (developmental) processes involved in defense/senescence. These included a cc-NB-type disease resistance protein (SOLYC12G044200), an RLK (SOLYC09G007110), WD40 domain-containing proteins (protein interactions, SOLYC11G010910 and SOLYC12G035360), MADS-box gene (jointless, which regulates abscission, SOLYC11G010570), and several F-box proteins involved in turnover (SOLYC09G005480, SOLYC11G011230, and SOLYC08G082510). Other key genes appeared to be involved in gene expression/regulation (containing B3 or BSD domains), protein (methyl transferase, glycosyltransferase, and phosphodiesterase), or lipid modification (Alg9-like) (see Supplementary Table S3).

Other genes, such as kinesin-like calmodulin-binding protein (SOLYC12G038430), proteasome subunit beta type-1 (SOLYC03G033740), and skin secretory protein xP2-like (SOLYC09G005430), were expressed nearly exclusively and to high levels in the resistant compared with the susceptible genotype. Overall, several of the most highly connected ‘hub’ genes identified in brown2 were related to the proteosome and F-box proteins, indicating linkage to protein turnover and cell death (Xu *et al.*, 2009); however, others require functional explanation (Supplementary Table S3).

In contrast, sample eigengene values in skyblue1 were consistently negative for the resistant genotype (and positive for the susceptible genotype), regardless of infection (Fig. 6A, B). Fifty-seven of the 74 genes had an annotation. Nine putative plant resistance genes were included and six of them contained nucleotide-binding domains [SOLYC12G044190 (Pvr4 homolog), SOLYC04G012010, SOLYC09G005290, SOLYC06G064720, SOLYC11G006630, and SOLYC10G055120]. Other types of resistance-associated genes, an RLK (SOLYC01G106500), a MAP kinase kinase 20 (SOLYC02G090430), and serine/threonine-protein kinase PBS1 (SOLYC02G094380), were included in this module.

Other genes in this module included tRNA (SOLYC12G062160), 50S ribosomal protein l23-like (SOLYC11G011140), 60S ribosomal protein L3 (SOLYC05G150151), the regulation of leaf senescence such as ethylene-insensitive protein 2 (SOLYC09G005490) and RING/U-box superfamily protein (SOLYC11G011570) (Zhang *et al.*, 2020), F box proteins (SOLYC11G005160, SOLYC11G012560), zinc finger BED domain-containing protein RICESLEEPER 2 (SOLYC04G057790 and SOLYC02G038727), and SelT-like proteins (SOLYC09G005580 and SOLYC09G005590).

Key hub genes included SelT-like (thioredoxin-disulfide reductase/chloroplast), Pvr4 (potyvirus resistance, cc-NB), and Homeobox-leucine zipper protein ROC5 (SOLYC06G035940, epidermis differentiation) (Supplementary Table S3). Overall, with few possible exceptions, no obvious functional classes of genes were found to be associated with genotype-specific gene expression.

### Physical clustering and gene expression patterns of genotype-associated genes

**Fig. 8.**
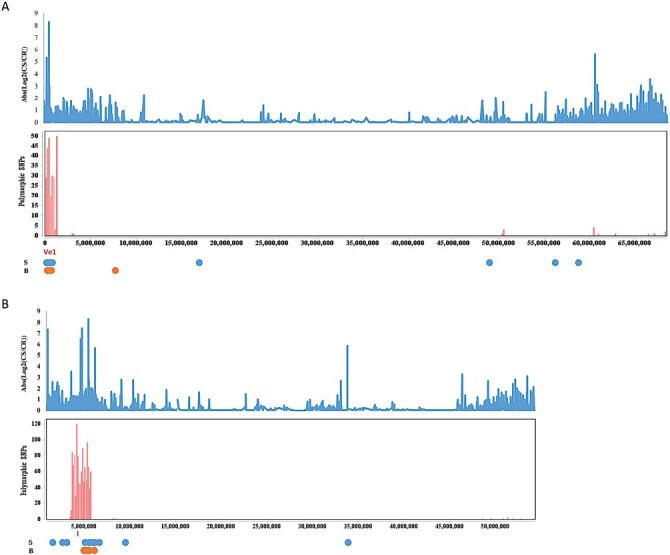
Comparison of gene expression and polymorphism differences along chromosomes 9 (A) and 11 (B) in NILs. Blue bars indicate gene clusters and regions showing large gene expression differences. Red bars indicate the number of polymorphism differences between NILs against the reference tomato genome. S=genes in module skyblue1, B=genes in module brown2. The locations of *Ve1* and *I* genes are indicated.

To gain further insight into *Ve1* resistance and the possible significance of genotype-specific gene expression patterns, we explored additional features of these genes. We evaluated the physical location of genes in modules brown2 and skyblue1 and examined gene polymorphisms in the context of expression patterns across chromosomes. The 115 brown2 and skyblue1 module genes were located on all chromosomes, ranging from 4 to 22 per chromosome. However, genes on chromosomes 9 and 11 appeared to be non-randomly distributed (Fig. 8). Near the end of chromosome 9, a total of 10 genes were physically clustered (from SOLYC09G005290 to SOLYC09G006000), each separated by no more than 15 genes. Overall, the region spanned >70 genes. The *Ve1* gene (SOLYC09G005090) was located distally, right next to this cluster (Fig. 8A). Fifteen genes were physically clustered (from SOLYC11G010570 to SOLYC11G011920) on chromosome 11, spread by no more than 23 genes, spanning ~135 genes (Fig. 8B). To examine patterns in gene expression, we determined the absolute difference in read count for non-infected rootstocks between the *ve1* and *Ve1* lines across the chromosomes. Visual inspection revealed that there was a strong positive correlation for differences in gene expression between the two lines and these gene cluster regions on both chromosomes 9 and 11 (Fig. 8). Evaluation of genome polymorphisms (SNPs) revealed polymorphic blocks only on chromosomes 9 and 11 (Fig. 8). The high polymorphic area at the end of the short arm of chromosome 9 covered 1.35 Mb and included the *Ve1* locus. On chromosome 11, the high polymorphic area spanned 2.18 Mb and also embraced the region that showed strong gene expression differences between the lines. SOLYC11G011180, the *I*-gene, which confers resistance to race 1 of *Fol*, was located in the middle of this region (Heisey, 2015). In sum, we observed that many (but not all) genes associated with genotype differences (based on expression) were physically clustered, with one cluster including the *Ve1* gene itself, and that this region was polymorphic between the two NILs. The significance of these observations is discussed.

### Expression of pathogen receptor genes (PRGs)

To explore genes associated with infection to *V. dahliae* in more depth, we identified 1172 genes expressed in this study which were classified as PRGs (http://prgdb.org/prgdb/) (Table 1). About 32% of these genes were included in the turquoise module, representing 9.4% of genes in this module, indicating that they were up-regulated during infection. Further, 11% of PRGs were included in the mediumpurple3 module, indicating that they were down-regulated during infection. Altogether, we found that 43% of these PRGs expressed in tomato were related to infection. This is in contrast to the 1% and 10% of genes related to the *Ve1*(+/–) plant genotype in modules brown2 and skyblue1 and scion/rootstock plant tissue type in modules black and orange, respectively.

**Table 1.** Expression of pathogen receptor genes (PRGs) in the trait-related modules

R class	SL4	Expressed	Turquoise	Mediumpurple3	Brown2	Skyblue1	Black	Orange
CK	86	74	16	15	0	1	3	0
NB	322	190	67	15	1	6^*^	21	6
KIN	776	566	177	80	1	1	34	12
RLK	233	197	43	16	1	1	25	5
RLP	167	101	49^*^	6	0	0	9	0
Others	83	44	19	2	0	0	2	0
Total no. of R genes	1667	1172	371	134	3	9	94	23
Total no. of genes	34 075	22 312	3958	3128	41	74	2333	626

^*^Indicates the significant enrichment based on the F exact test in *P*<0.05.

Fisher’s exact test revealed that the RLP-type defense genes were the most significantly over-represented class. Among the 101 expressed RLP genes, almost half—49 genes—were included in module turquoise and six in module mediumpurple3 (Table 1). R genes containing a nucleotide-binding site (NB) were over-represented in the module skyblue1. NB-type R genes containing an NB domain are typically predicted to be cytosolic. The six NB-type defense genes, SOLYC04G012010, SOLYC06G064720, SOLYC09G005290, SOLYC10G055120, SOLYC11G006630, and SOLYC12G044190, showed 2–19 times increased expression in the susceptible rootstock compared with the resistant rootstock, regardless of infection. These data indicate that infection resulted in dramatic changes in the expression of a disproportionate number of genes commonly associated with pathogen detection and defense, particularly those encoding membrane receptor-like proteins.

### Clustering and co-expression of *Ve1* and other pathogen receptor genes

**Fig. 9.**
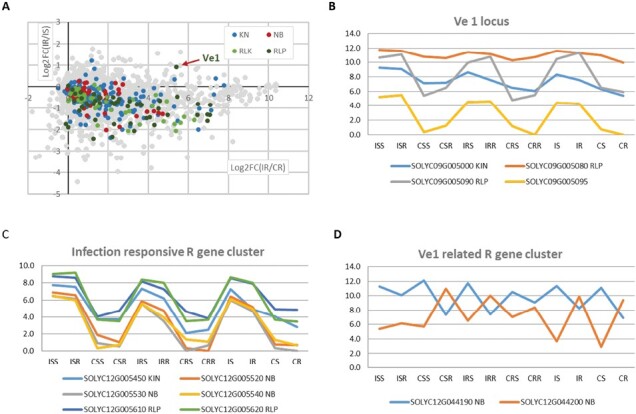
Expression of pathogen receptor genes (PRGs). (A) Differential gene expression during infection was calculated by the log2 fold changes of genes between IR and CR samples [logFC(IR/CR)] and plotted with the differential gene expression between infected susceptible and resistant rootstocks [log2FC(IR/IS)]. Groups of PRGs were color-coded and the gray color represents the non-PRGs. Expression patterns of clustered genes in the *Ve1* locus (B), infection-responsive R gene cluster (C), and genotype-linked R gene cluster (D) are shown by the normalized gene read count over each sample.

A majority of PRGs that were induced during infection (mostly included in the module turquoise) tended to be more highly expressed in susceptible than in resistant rootstock tissue (Fig. 9A). A primary exception was the *Ve1* gene, which although highly induced upon fungal infection tended to be more induced in the resistant tissues than in the susceptible tissues. A strong induction during infection was also found in the *Ve1* neighboring gene, SOLYC09G005095 (unknown function). *Ve2* (SOLYC09G005080), a *Ve1* paralog also located next to *Ve1*, showed a relatively stable gene expression pattern with a slight increase of expression upon infection and no difference between susceptible and resistant tissues. The neighboring lectin receptor-like kinase, SOLYC09G005000, showed a similar expression pattern to *Ve2* (Fig. 9B).

*Ve1* was strongly co-expressed with the hub genes in the module turquoise including Cf2/5 homolog (SOLYC12G099980), Cf4/9 homolog (SOLYC01G009690), ATPases (SOLYC03G033840 and SOLYC03G033790), MYB transcription factors (SOLYC03G093890 and SOLYC03G093930), prenylated Rab acceptor protein 1 (PRA1) family protein (SOLYC01G089890), C2 Ca^2+^-dependent membrane-targeting protein (SOLYC08G080530), and peroxisomal membrane protein PMP22 (SOLYC06G053550) (Fig. 7).

We found that many of the RLPs and RLKs induced during infection (in the module turquoise) were associated with clusters of PRGs across different chromosomes, typically found near chromosome ends (Supplementary Fig. S2). Certain clusters such as at the end of chromosome 12 also included protein kinase and NB proteins in addition to RLPs (Fig. 9C).

Another observation of note was the opposite expression pattern of a pair of co-located NB-type resistance genes SOLYC12G044190 and SOLYC12G044200. These two genes showed very strong sequence similarity and are homologs of the Pvr 4 disease resistance gene of pepper (Kim *et al.*, 2017). SOLYC12G044190 was more highly expressed in the susceptible genotype (regardless of infection) compared with the resistant line, and was a member of the skyblue1 module. In contrast, SOLYC12G044200 showed the opposite pattern and was a member of brown2 (Fig. 9D).

### Fungal gene expression in the *V. dahliae*-infected tomato tissue

**Fig. 10.**
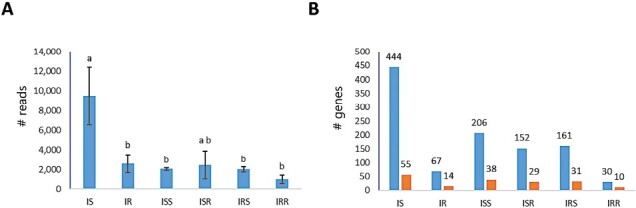
*Verticillium dahliae* gene expression *in planta*. (A) The number of reads mapped to the VdLs 17 genome. (B) The number of *V. dahliae* genes (blue) and genes with signal peptide (orange) identified in tomato samples. At least one read was mapped to the gene in all biological replicates.

Compared with plant gene expression, detection of fungal gene expression in infected tissue samples was very low. An average of <10 000 reads mapped to *V. dahliae* in the rootstock of the susceptible line (IS), which was significantly more than found in the resistant rootstock (IR). Gene expression levels mirrored the relative amounts of fungal biomass in each tissue, with significantly more fungus present in the IS tissues compared with other tissues as determined by qPCR (Supplementary Fig. S3). The higher fungal read number in IS resulted in mapping to 444 genes, compared with 67 genes in IR. No significant trends were found between scion treatments; each registered no more than 3000 reads; however, the resistant scion on the resistant rootstock yielded the fewest fungal reads. Genes with signal peptides represented 12–33% of the identified genes across the fungal samples (Fig. 10).

Functional analysis of fungal expressed genes in the infected rootstock of the susceptible rootstock (IS) showed that genes for primary metabolism, translation, and energy production were abundant (Supplementary Fig. S4). Also, genes involved in response to oxidative stress were abundant and included cytochrome *c* peroxidase (VDAG_03116), ligninase H8 (VDAG_06204), peroxidase/catalase (VDAG_02834 and VDAG_04826), and peroxiredoxin HYR1 (VDAG_08498). We also identified the presence of salicylate hydroxylase (VDAG_04960, VDAG_07329), which regulates SA levels (Ambrose *et al.*, 2015).

For a more in-depth analysis of genes possibly involved in pathogenesis, we evaluated the most highly expressed genes in the susceptible rootstock (IS) that contained a signal peptide (Table 2). Among the top 20 genes, 11 genes had a signal peptide, including the most highly expressed gene, VDAG_10515. The VDAG_10515 homolog, VnaSSP4.2, works as a virulence factor and is required for *V. nonalfalfae* colonization in hop plants (Flajsman *et al.*, 2016).

**Table 2.** Top 40 most highly expressed fungal genes in infected tissues

Gene ID	Annotation	AA	IS	IR	ISS	IRS	ISR	IRR	SP
VDAG_10515	Hypothetical protein	127	238	38	82	84	58	41	Yes
VDAG_09492	2-Oxoglutarate-dependent ethylene/succinate-forming enzyme	377	154	10	24	19	17	5	
VDAG_01109	Hypothetical protein	361	94	10	14	12	30	7	Yes
VDAG_05456	Pisatin demethylase	497	90	26	10	16	39	4	
VDAG_08142	Hypothetical protein	595	83	16	12	18	21	6	
VDAG_10367	Hypothetical protein	294	81	20	13	20	16	9	Yes
VDAG_07646	Acetamidase	546	79	14	16	13	15	7	
VDAG_07185	Glucan 1,3-β-glucosidase	357	66	19	14	19	15	9	Yes
VDAG_09574	Hypothetical protein	490	63	6	14	18	8	9	Yes
VDAG_01167	Multidrug resistance protein CDR1	1517	62	36	14	10	23	7	
VDAG_04336	α-*N*-Arabinofuranosidase	509	61	25	11	11	18	5	Yes
VDAG_07881	Pectinesterase	332	57	8	13	10	10	7	Yes
VDAG_06133	Amine oxidase	453	56	18	10	10	13	3	Yes
VDAG_07771	Oxidoreductase	298	53	10	8	13	3	6	
VDAG_09846	Hypothetical protein	496	52	20	7	13	9	12	Yes
VDAG_09510	Glucan 1,3-β-glucosidase	519	49	19	12	9	15	3	Yes
VDAG_01315	Formate dehydrogenase	348	48	7	8	9	5	5	
VDAG_08653	Hypothetical protein	154	47	15	5	9	9	5	Yes
VDAG_02559	Elongation factor 1-α	473	43	14	8	11	10	2	
VDAG_08639	Hypothetical protein	135	41	12	5	8	15	2	
VDAG_05343	Hypothetical protein	592	40	6	7	5	11	2	
VDAG_02913	Brefeldin A resistance protein	1409	37	15	7	7	8	1	
VDAG_08101	Aldehyde dehydrogenase	482	37	18	5	8	12	1	
VDAG_04957	3-Ketoacyl-CoA thiolase	421	36	1	9	11	6	0	
VDAG_02735	Hypothetical protein	155	35	15	6	8	9	7	Yes
VDAG_00915	Hypothetical protein	130	34	2	6	7	3	3	
VDAG_01837	Metallo-β-lactamase superfamily protein	309	31	10	7	9	13	3	
VDAG_09991	Quinate permease	559	30	8	9	10	6	4	
VDAG_06662	Hypothetical protein	65	30	5	7	6	7	3	
VDAG_07680	Hypothetical protein	224	29	4	4	1	7	3	
VDAG_08141	Choline dehydrogenase	612	29	6	7	14	4	3	Yes
VDAG_06722	Monodehydroascorbate reductase	548	28	13	4	6	14	2	
VDAG_02256	Hypothetical protein	134	25	4	1	3	2	3	
VDAG_07316	Homogentisate 1,2-dioxygenase	448	25	7	4	3	6	2	
VDAG_01905	Hypothetical protein	159	25	4	5	3	6	4	Yes
VDAG_08026	ATP-dependent permease PDR10	1469	24	2	7	4	1	0	
VDAG_07681	ATP-binding cassette sub-family G member 5	625	24	5	10	8	3	6	
VDAG_03325	Cupin domain-containing protein	191	24	11	5	6	5	1	
VDAG_00200	Medium-chain specific acyl-CoA dehydrogenase	566	23	10	6	3	9	4	
VDAG_02834	Peroxidase/catalase	762	23	4	9	9	5	1	

Values represent average reads for three replicates. AA indicates an encoded number of amino acids. The presence of a signal septide (SP) is indicated. See Fig. 1 for sample codes.

Other highly expressed genes included plant defense detoxifying enzymes, such as pisatin demethylase homolog (VDAG_05456), cell wall-degrading enzymes, pectinesterase (VDAG_07881) and glucan 1,3-beta-glucosidase (VDAG_07185, VDAG_09510), and oxidative stress-responsive proteins oxidoreductase (VDAG_07771) and peroxidase/catalase (VDAG_02834). Many membrane transporters were highly expressed including a multidrug resistance protein CDR1 (VDAG_01167), a brefeldin A resistance protein (VDAG_02913), quinate permease (VDAG_09991), and an ATP-dependent permease PDR10 (VDAG_08026).

In a previous study, we predicted effector proteins, some of which were lineage specific (Ingram *et al.*, 2020). In this study, we found that several were highly expressed (Supplementary Table S5). Most of these were annotated as hypothetical proteins, but some homologs have been reported as virulence factors or related to virulence in other host/pathosystems. In addition to the VDAG_10515 homolog reported to be involved in virulence in the hop-infecting *V. nonalfalfae* strain (Flajsman *et al.*, 2016), we identified VDAG_10367 as a homolog of the endoglucanase C in *Fusarium heterosporium* and VDAG_02735 as a known virulence factor PevD1 in the cotton-infecting *V. dahlia* strain (Zhang *et al.*, 2019). Furthermore, when sequence reads were mapped to strain JR2, we found the two known avirulence effectors, VdAve1 and Av2, which were expressed in IS with an average of 15 and 18 mapped reads, respectively (De Jonge *et al.*, 2012; Chavarro-Carrero *et al.*, 2021).

## Discussion

### *Ve1* gene confers resistance to scions independent of the rootstock genotype

Here, we used bi-grafted planFts of tomato NILs coupled with analysis of gene expression patterns to provide comprehensive insight into defensive responses of resistant and susceptible tissues to *V. dahliae* race 1. In our hands, examining both reciprocal and bi-grafted plants of the Craigella NILs, wilting and typical lower leaf lesion symptoms only developed in the susceptible scions, whatever the genotype of the rootstock, within a few weeks after infection (Fig. 2). Rootstock did not affect the time or extent of symptom appearance. Symptoms were not observed in resistant scions on susceptible rootstocks, even 6 weeks after infection. Studies by others using the same reciprocally grafted NILs yielded different results. Nazar *et al.* (2018) reported that resistant rootstock plants conferred a healthy phenotype to the scion, whereas scions on susceptible rootstock plants wilted 10 DAI (Nazar *et al.*, 2018). Chen *et al.* (2004), found that the fungus enters the xylem and moves through the vasculature into the upper tissues of both susceptible and resistant plants within a few days (Chen *et al.*, 2004). It is only later that proliferation continues in susceptible plants, whereas pathogen levels fluctuate, but remain low in the resistant plants, suggesting that resistance is conveyed by the upper foliar tissues. Perhaps differences in the experimental conditions provide a clue to the apparent discrepancy with the work of Nazar *et al.* (2018) as we did not observe wilting symptoms by 10 DAI. Wilting by 10 DAI may indicate that the vascular system of the susceptible rootstock had been extensively invaded and is unable to support water movement to the scion tissues (Nazar *et al.*, 2018). Also, it is not uncommon to observe early wilting of grafted plants after infection, which eventually recover at later time points.

In the field, grafted plants, including tomato, have been used effectively to manage other wilt pathogens including *Fol* and *R. solanacearum* in many cases (Louws *et al.*, 2010; Thies, 2021). However, for *V. dahliae* resistance in tomato, the use of grafted plants has not been extensively deployed. Indeed, rootstocks of resistant lines have often shown little to no benefit to scions of susceptible varieties (Louws *et al.*, 2010; Acharya *et al.*, 2020; Thies, 2021). These observations support our laboratory studies showing that resistance to *V. dahliae* is not conferred by the rootstock.

### Increased defense response in the susceptible tomato line

In our studies, we observed dramatic changes in gene expression 10 DAI of the NILs of tomato with race 1 *V. dahliae* in both the resistant (*Ve1*) interaction and the susceptible (*ve1*) interaction. Based on DEG analyses, up to 15% of the genome of rootstock and scions responded to *V. dahliae* infection (Fig. 5). However, overall, we found no apparent differences in gene expression patterns in scions or rootstocks between resistant and susceptible plants, although susceptible tissues responded to a greater extent, with ~25–35% more DEGs. Interestingly, we found that many infection-induced genes related to plant defense genes were induced to a lesser degree in resistant tissue compared with susceptible tissue. The more elevated expression of PR and other defense genes in the susceptible tissue was previously reported using the same susceptible and resistant lines utilizing small-scale microarray experiments and confirmed by protein levels (Robb *et al.*, 2009, 2012) and also during a tolerance response against *V. dahliae* infection (Robb *et al.*, 2012).

Conclusions from other studies examining gene expression in resistant versus susceptible interactions for other wilt-causing pathogens in tomato or Verticillium in other crops largely differ from observations of *V. dahliae* resistance in tomato (Xu *et al.*, 2011; Manzo *et al.*, 2016; Chang *et al.*, 2021). Generally, other studies support the hypothesis that resistance is seen as a more rapid and robust expression of defense mechanisms. In the case of tomato and *V. dahliae* race 1 resistance, we do not know how the apparent reduced level of defense response is linked to resistance conferred by *Ve1*. As suggested by Robb *et al.* (2012), hyperactivation of defense-related genes, working as susceptibility factors, could result in cell death that eventually manifests as typical Verticillium disease symptoms such as chlorosis and yellowing that we found only in the susceptible scions in this study. It is also feasible that resistance is highly localized to the cells surrounding the xylem vessels as has been reported for I-2 *Fol* in tomato (Mes *et al.*, 2000). As such, a specific resistance response may have been masked by the bulk of surrounding parenchyma cells. We did also observe small differences in DEGs between susceptible and resistant in the rootstocks (Fig. 3B), and we also noted that the resistant rootstock slightly influenced gene expression in the scion in response to *V. dahliae* (Fig. 4A). These observations suggest the rootstock (roots and hypocotyl tissues) may have some role in *V. dahliae* resistance.

### Role of cell surface receptors in *Ve1*-mediated downstream signaling pathways

Race-specific resistance to *V. dahliae* race 1 is dependent on the co-existence of Ve1, an RLP, in tomato and virulence effector VdAve1 in *V. dahliae* strains (Kawchuk *et al.*, 2001; De Jonge *et al.*, 2012). Of note, we observed that *Ve1* was strongly induced upon infection and elevated (~2-fold) more in the resistant than in the susceptible line (Fig. 9A). Fradin *et al.* (2009) also observed that the peak of *Ve1* expression occurred slightly earlier in the resistant line. How membrane signals are transferred to activate cellular defense remains to be fully elucidated. Defense signaling responses to *V. dahliae* and to leaf mold of tomato caused by *Cladosporium fulvum* both require both BAK1 and SOBIR (Fradin *et al.*, 2009, 2011; Liebrand *et al.*, 2013; Albert *et al.*, 2015). However, we observed that expression of neither gene was more highly elevated in the resistant interaction.

Ve1 alone may not fully explain resistance or our observations that susceptible tissues evoked a more dramatic defense gene expression than resistant tissues. Transgenic VdAve1 expression in the susceptible tomato line (*ve1*, LA3247) also induced various defense genes, suggesting that other receptor proteins, possibly Ve2, might be involved in defense signal transduction (Castroverde *et al.*, 2016). Recently, Kalischuk *et al.* (2022) showed that *Ve1* and *Ve2* independently recognized Verticillium infection in transgenic potato plants and collaborate to induce disease resistance by the formation of the *Ve1Ve2* heteromeric receptor complex. In our study, similar to previously work (Castroverde *et al.*, 2017), *Ve2* was constitutively highly expressed in resistant and susceptible lines (Fig. 9B).

Further, we found many other RLP receptors were enriched upon *V. dahliae* infection, with nearly 50% of annotated RLPs responding to infection (in module turquoise) (see Table 1). They included *Ve1* and many Cf2/5 and Cf4/9 homologs clustered on chromosomes 1 and 12 related to recognition of the foliar pathogen *C. fulvum* (Jones *et al.*, 1994; Dixon *et al.*, 1996, 1998; Thomas *et al.*, 1997) as well as non-host-specific defense proteins LeEIX1 and LeEIX2 that mediate recognition of the ethylene-inducing xylanase of *Trichoderma viride* (Ron and Avni, 2004).

Most RLPs are known to be expressed differentially according to tissue type, development, and response to various abiotic and biotic stresses (Kang and Yeom, 2018). For example, analysis of tomato response to bacterial infection with *Pseudomonas fluorescens* and *P. syringae* DC-3000 revealed that five RLPs were commonly up-regulated, while 16 were more highly induced by *P. fluorescens* and six by *P. syringae* DC-3000 infection. Of these genes, the vast majority were induced in our study. In contrast, of the 19 RLPs up-regulated in the susceptible reaction to the tomato yellow leaf curl virus, few were found to respond to *V. dahliae* infection (Kang and Yeom, 2018). Nazar *et al.* (2019) also reported that the *Ve1* expression was induced by wounding, but not by salinity or water deficit (Nazar *et al.*, 2019). Overall, this suggests particular groups of RLPs are induced to potentially combat different pathogens.

### Role of NB defense responses in *Ve1* resistance

Recently the synergic effects for plant immunity between PTI and ETI through the collaboration of membrane and intracellular receptors have been reported (Ngou *et al.*, 2021; Yuan *et al.*, 2021). In our work, we noted that the expression of many NB proteins was altered due to *V. dahliae* infection (see Table 1); however, unlike RLPs, network analysis revealed that their expression patterns were not highly connected. To date, few have been fully characterized, notably Bs4 which confers resistance to bacterial spot (Schornack *et al.*, 2004), Hero to yellow potato cyst nematode (Ernst *et al.*, 2002), Tm-2 to tobacco mosaic virus (Lanfermeijer *et al.*, 2003), and Sw-5 to tomato spotted wilt virus (Zhu *et al.*, 2017). Intriguingly, resistance to *Fol* race 2 is encoded by a cytosolic NB gene I-2 (Ori *et al.*, 1997; Mes *et al.*, 2000). Many of these NB proteins are considered sensor receptors that typically interact with other NB proteins, referred to as helper NBs, to activate defense pathways and HR (Bonardi *et al.*, 2011). The helper NB, NRC1 (NB-LRR Required for Hypersensitive Response-Associated Cell Death-1) is required for the *Ve1*-mediated resistance (Fradin *et al.*, 2009) as well as resistance to other pathogens (Gabriëls *et al.*, 2007; Chen *et al.*, 2021). Other NB receptors, GbRVd, GbaNA1, and GbCNL130, confer resistance to Verticillium wilt in cotton (Yang *et al.*, 2016; Li *et al.*, 2018, 2021). EDS1 (Enhanced Disease Susceptibility), which is considered to mediate NB defense signaling pathways, is also required for *Ve1*-mediated resistance. In this study, we found that four helper NBs (SOLYC01G090430, SOLYC04G007070, SOLYC05G009630, and SOLYC10G047320) in tomato were constitutively and highly expressed. Moreover, GbRVd and GbaNA1 homologs were significantly induced upon infection. A pair of NB genes (SOLYC12G044190 and SOLYC12G044200) were unusual as they were differentially expressed with opposing patterns in the susceptible and resistant tomato lines (Fig. 9D). The significance of this observation remains to be determined. Further, the signaling connections between RLPs and NB proteins with regard to effector-mediated defense remain obscure. Of note, several RLP and NB genes were physically clustered and showed similar expression patterns (Fig. 9C). This observation may have functional significance where lineage conservation of gene blocks favors possible cooperation among these receptors in response to pathogen attack. Further functional characterization of *Ve1*-related intracellular receptor proteins may shed further insight into *V. dahliae* resistance.

### Other key insights into tomato responses to *Verticillium* infection

In addition to several RLPs, we discovered genes belonging to the AAA-ATPase At3g50940-like superfamily to be key network hubs in response to *V. dahliae* infection (Fig. 7). These enzymes have been associated with diverse biological functions including proteolysis, morphogenesis, and leaf senescence (Snider and Houry, 2008; Yedidi *et al.*, 2017). In a few instances, AAA-ATPase genes from *N. tabacum* (*NtAAA1*), Arabidopsis (*AtOM66*), and rice have been shown to be involved in the SA signaling pathway and the HR upon pathogen infection (Liu *et al.*, 2020). A key feature of the family is the presence of a ring-shaped P-loop structure bearing ATP hydrolytic function. The P loop is a common feature of many NB proteins including helper NBs such as NRC1 (Jubic *et al.*, 2019). Defense signaling activity for NRC1 is dependent on the P loop (Sueldo *et al.*, 2015). Thus, it is tempting to suggest that these genes serve overlapping functions with NB proteins and deserve further examination.

As mentioned in the Introduction, defense responses to xylem-infecting pathogens appear to be multilayered. Our results showed that several chitinase genes and subtilisin-like proteases were strongly up-regulated and were highly connected in response to *V. dahliae* infection (Fig. 7). These enzymes are considered core components of the PR or pathogenesis-related proteins (Linthorst and Van Loon, 1991). Both groups of enzymes have been extensively studied in the context of host defense gene activation. Recognition of chitin fragments, released through the action of host chitinases, by membrane-associated receptors such as Cf4 represent a common and key strategy for defense against fungal pathogens (Linthorst and Van Loon, 1991). Likewise, subtilases are transcriptionally activated in response to numerous wounding/biotic stresses, including in tomato (Figueiredo *et al.*, 2018). Several of these enzymes have been shown to accumulate in the extracellular matrix and stem walls (Dahal *et al.*, 2010; Ramírez *et al.*, 2013). Functional analyses utilizing gene knockdown have confirmed the importance of subtilases in resistance, including against *V. dahliae* in cotton (Duan *et al.*, 2016).

Activation of PR genes has been reported throughout the entire plant, involving regulation (phosphorylation) of SA-, ethylene-, JA-, and abscisic acid-mediated signaling pathways that results in systemic acquired resistance (Vlot *et al.*, 2021; Zhou *et al.*, 2021). Given the low colonization of the fungus (based on read counts) detected in infected tissues in our study, the extent of host responses is particularly impressive. One possible explanation may be due/related to continuous and persistent irritation of the roots by the pathogen that triggers systematic responses throughout the entire plant.

We found that transcription factors including MYB, WRKY, and ethylene-responsive transcription factors were strongly induced during *V. dahliae* infection. These included several WRKY transcription factors that were previously reported to be up-regulated under the stress conditions from drought, salt, and bacterial infection (Huang *et al.*, 2012; Bai *et al.*, 2018). Detailed analyses of the defense response against *V. dahliae* in *Arabidopsis thaliana* by Su *et al.* (2018) uncovered a complex signaling network involving a myriad of different transcription factors.

Genes related to cell wall and membrane modifications were identified as infection-related hub genes. In the case of xylem-inhabiting pathogens, structural and other changes associated with the vascular system have long been proposed to be associated with resistance to *V. dahliae* and other wilt-inducing pathogens (De Coninck *et al.*, 2015; Kashyap *et al.*, 2021). Homologs of R2R3MYB transcription factors SOLYC03G093890 and SOLYC03G005570, AtMYB58, and AtMYB15 were shown in Arabidopsis to activate genes involved in lignin biosynthesis pathways including PAL (Zhou *et al.*, 2009; Kim *et al.*, 2020). We also found several rate-limiting genes in the phenylpropanoid pathway including PAL, cinnamyl alcohol reductase, and several peroxidases induced by *V. dahliae* infection. Likewise, key genes in suberin formation, including tyramine hydroxycinnamyl transferase, tyramine *N*-feruloyl transferase, and fatty acid metabolism were induced. Several fatty acid desaturases were highly elevated by *V. dahliae* infection, which has been linked to pathogen defense, potentially through structural modifications to membranes as well as via increased biosynthesis of oxylipins, particularly JA (Laureano *et al.*, 2021).

However, based on our analyses of whole tissue, we observed little difference in gene expression patterns between resistant and susceptible lines. More detailed studies including tissue localization are needed to dissect the role of defense receptors, signaling genes, PR proteins, and structural wall modifications more precisely in restricting *V. dahliae* growth in the vasculature of resistant tissues.

### Unforeseen genotype-dependent gene expression pattern differences

To minimize differences in gene expression due to genetic background, we used the near-isogenic plants Craigella (LA3247, susceptible) and a Craigella line into which had been introgressed the *Ve1* gene (LA3428, resistant). We did observe a small number of genes differentially expressed between these lines under the non-infected conditions. Most of these genes were included in modules brown2 and skyblue1 (Fig. 6). These genes may be involved in determining *V. dahliae* disease outcome to race 1. However, no obvious enrichment of genes involved in pre-formed changes in cell wall modifications such as suberin or lignification was apparent, suggesting no large-scale structural changes between the near-isogenic plants. Interestingly, we found that many of these genes were physically clustered, with one of the clusters being linked to the *Ve1* locus itself (Fig. 8). Another cluster was prominent on chromosome 11. Inspection of the pedigree of the Craigella NILs revealed that LA3428 in addition to possessing the *Ve1* gene was generated to contain the *I* gene for resistance to *Fol*. This gene was indeed located in the middle of the cluster on chromosome 11. Further inspection of these chromosomes revealed the *Ve1* and *I* gene regions were rich in DNA polymorphisms. This suggests that even though LA3247 and LA3428 are thought of as near isogenic, there are likely to be significant background genetic variations around the *Ve1* and *I* loci as a consequence of linkage drag. NILs used here are estimated to have theoretical 97% recovery of the recurrent parent in the absence of linkage (Smith and Ritchie, 1983). Further study is needed to determine if and how genetic variations in these clusters affect *Ve1*-dependent resistance. The exploitation of plant lines where the *Ve1* gene has been specifically deleted or added will probably be of value (Nazar *et al.*, 2020).

### *In planta* effector gene expression

Due to low abundance of fungal mycelia in infected tissue, currently no comprehensive *in planta* analyses of *V. dahliae* have been reported (Faino *et al.*, 2012; Hu *et al.*, 2019). Instead, the analysis of gene expression under nitrogen starvation or microsclerotia development and the secreted protein profiles has provided some insight into *V. dahliae* pathogenicity (Xiong *et al.*, 2014, 2015; Chen *et al.*, 2016). In this study, in an attempt to overcome these limitations, we performed deep RNA-seq. However, even in susceptible rootstock hypocotyl tissue, <15 000 (~0.02%) of total reads mapped to the *V. dahliae* genome (Fig. 10). This low read number as well as qPCR evaluation of fungal DNA suggests that there was little fungal accumulation in the xylem tissues when the sample was collected 10 DAI. Although the data are limited, the gene expression profiles suggest that *V. dahliae* has means to cope with oxidative stresses, manipulating the host defense reactions (toxin degradation, SA, and ethylene), actively degrading cell wall components (pectinases and glucosidase), and metabolizing host carbon sources (such as quinate) to produce energy for survival and colonization in the host tissues (see Table 2). Many of the more highly expressed secreted proteins were hypothetical proteins; however, others have been characterized as virulence or avirulence factors. Currently, two avirulence genes *VdAve1* and *Av2* have been characterized in tomato (De Jonge *et al.*, 2012; Chavarro-Carrero *et al.*, 2021). Both of these genes are located in the lineage-specific chromosomal regions of the *V. dahliae* Le1087 genome (Ingram *et al.*, 2020) and were expressed in the plant scion tissues as well as rootstocks in this study. Others bore hallmarks of candidate effectors, which matched homologs in other fungal pathogens. These findings suggest that the analysis of highly expressed *in planta* secreted proteins from *V. dahliae* isolates from different genetic backgrounds will probably provide comprehensive pools of effector candidates that may be useful for new race prediction, monitoring population dynamics, and efficient disease management.

### Conclusions

Based on our data and the forgoing discussion, the relationship between tomato and the Verticillium wilt pathogen is complex. However, several conclusions can be drawn from this work: (i) based on symptom development, the scion confers resistance to Verticillium wilt race 1 in tomato, which is largely in contrast to defense against other soil-borne vascular pathogens; (ii) susceptible plants responded more dramatically to infection than resistant plants (based on gene expression); (iii) patterns of gene expression were generally similar in resistant and susceptible tissues; (iv) many different defense-related genes and pathways responded to infection, with receptor-like proteins being among the most enriched group of DEGs; (v) *Ve1* was more highly expressed in resistant tissues; (vi) rootstocks and scions exhibited different patterns of gene expression, which was primarily related to tissue physiology; (vii) genotype-specific blocks of genes were found to be differently expressed, which was probably related to linkage drag, although this requires further investigation; (viii) fungal gene expression was very low, but more genes and to a higher expression level were found in susceptible rootstocks, and included many secreted (candidate effector) genes; and (ix) network analysis revealed a multilayered and integrated response to *Verticillium* infection of tomato and provided a number of new key genes (hubs), whose role in defense responses needs further functional interrogation.

## Supplementary data

The following supplementary data are available at *JXB* online.

Table S1. RNA-seq data mapped to tomato and *V. dahliae.*

Table S2. Enriched GO terms in tomato-associated WGCNA modules.

Table S3. Tomato hub genes associated with WGCNA modules.

Table S4. Tomato annotated genes in WGCNA modules.

Table S5. Expressed *V. dahliae* effector genes.

Fig. S1. Symptoms and growth of bi-grafted tomato plants.

Fig. S2. Chromosome location and expression of RLPs.

Fig. S3. Fungal biomass measurement by quantitative real-time PCR.

Fig. S4. Annotation of *in planta V. dahliae* expressed genes.

erad182_suppl_Supplementary_Figures_S1-S4

erad182_suppl_Supplementary_Table_S1

erad182_suppl_Supplementary_Table_S2

erad182_suppl_Supplementary_Table_S3

erad182_suppl_Supplementary_Table_S4

erad182_suppl_Supplementary_Table_S5

## Data Availability

All data to support the conclusions of this study are included in the main text and the supplementary data. The RNA-seq data used in this study are deposited in the National Center for Biotechnology Information (NCBI) Sequence Read Archive (SRA) database https://www.ncbi.nlm.nih.gov/sra with the BioProject ID: PRJNA964766.
