# Supplementary material for: Tissues and mechanisms associated with Verticillium wilt resistance in tomato using bi-grafted near-isogenic lines
Source: J Exp Bot. 2023 May 15;74(15):4685–706. doi: 10.1093/jxb/erad182 (PMC10433936; doi:10.1093/jxb/erad182)
Supplement: erad182_suppl_Supplementary_Figures_S1-S4 [file erad182_suppl_supplementary_figures_s1-s4.pdf]

A

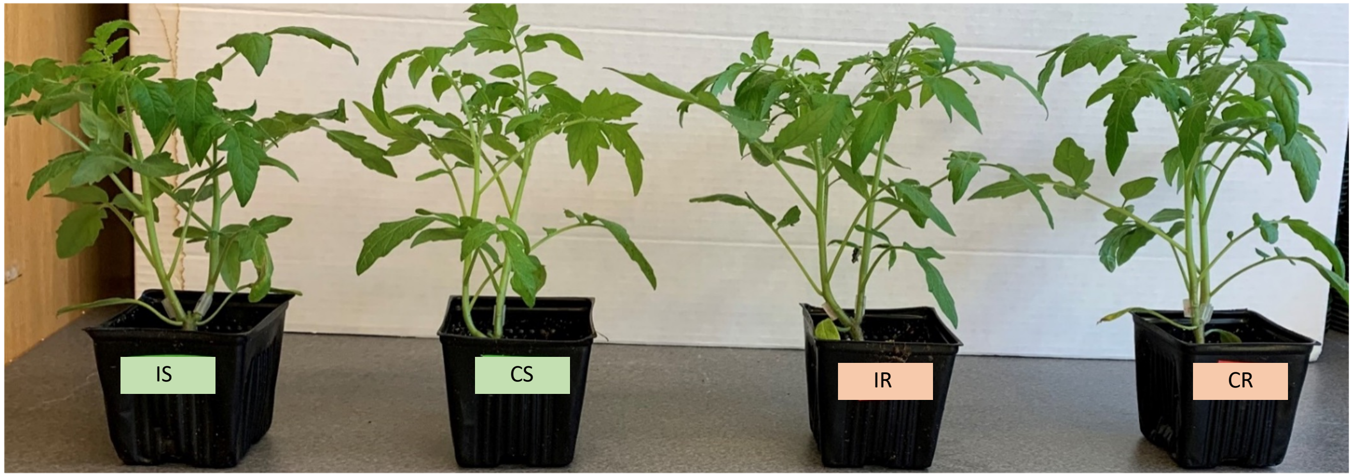

B

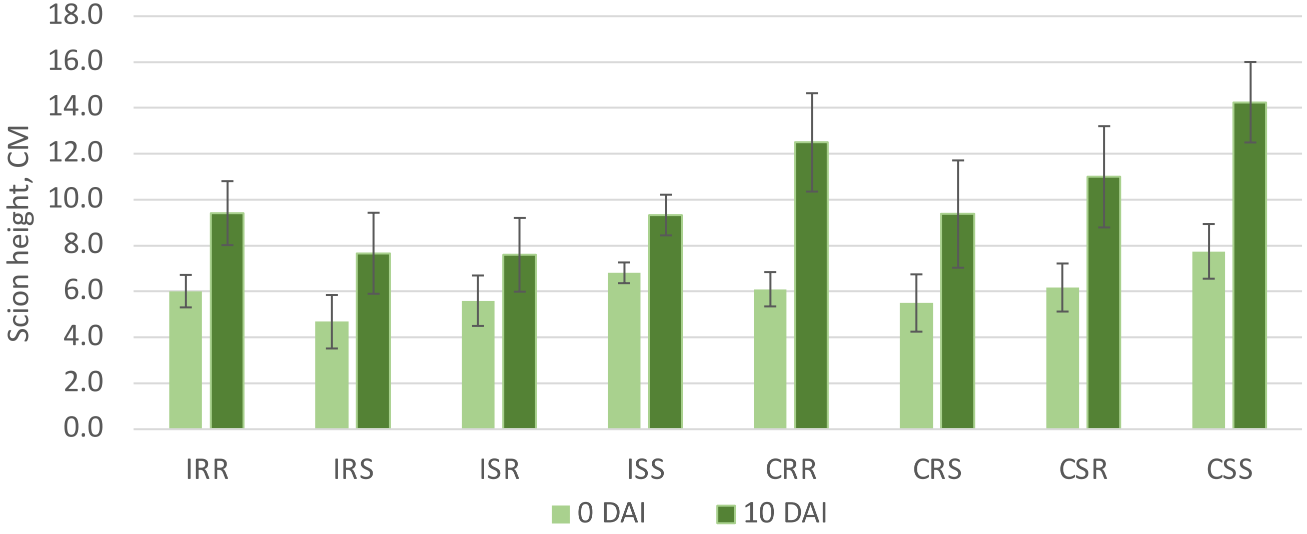

Supplementary Figure S1. *Disease symptoms and the plant growth of the bi-grafted plants at 0 and 10 DAI. Panel A. Photograph taken 10 DAI. Panel B. Plant height from point of graft to stem apex at 0 and 10DAI. See Figure 1 for sample codes.*

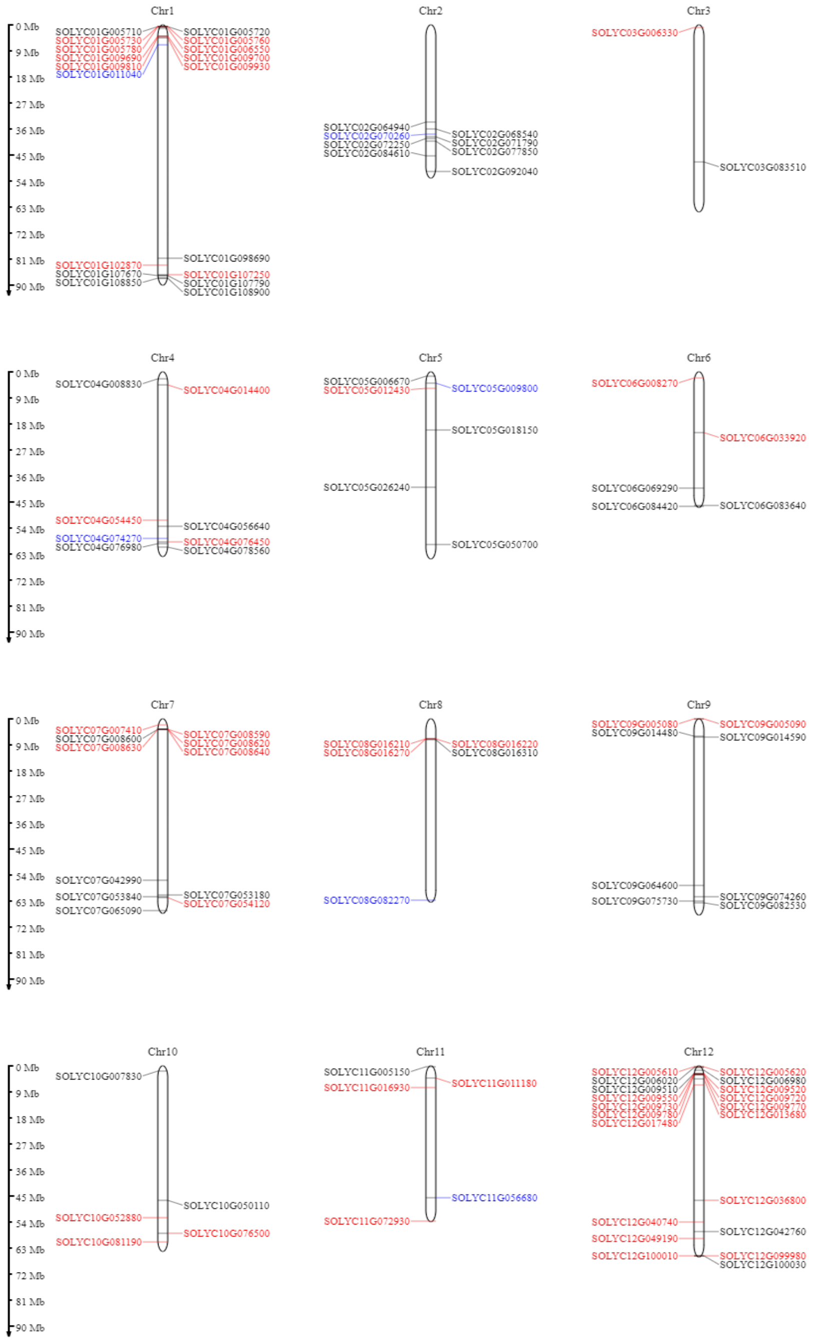

Supplementary Figure S2. Physical mapping of RLP genes in the tomato chromosomes. Genes included in the turquoise module are colored in red and genes for the mediumpurple3 are in blue.

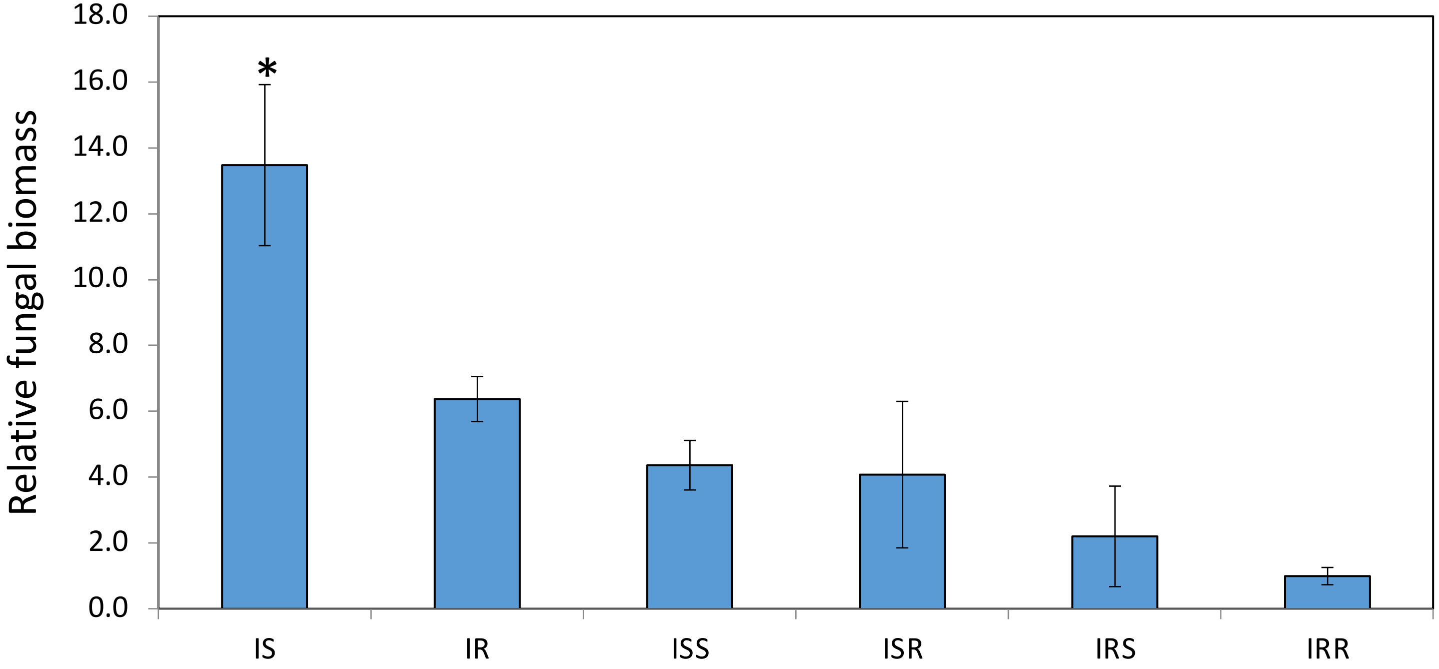

Supplementary Figure S3. Fungal biomass measurement by quantitative real-time PCR at 10 days after inoculation. Fungal biomass was plotted relative to that from resistant scion over resistant rootstock (IRR), which was set to 1.0. Bars represent mean values with standard deviation. The value of the column labeled with an asterisk differed significantly compared with those of other columns (Turkey-Kramer post hoc test, alpha = .01).

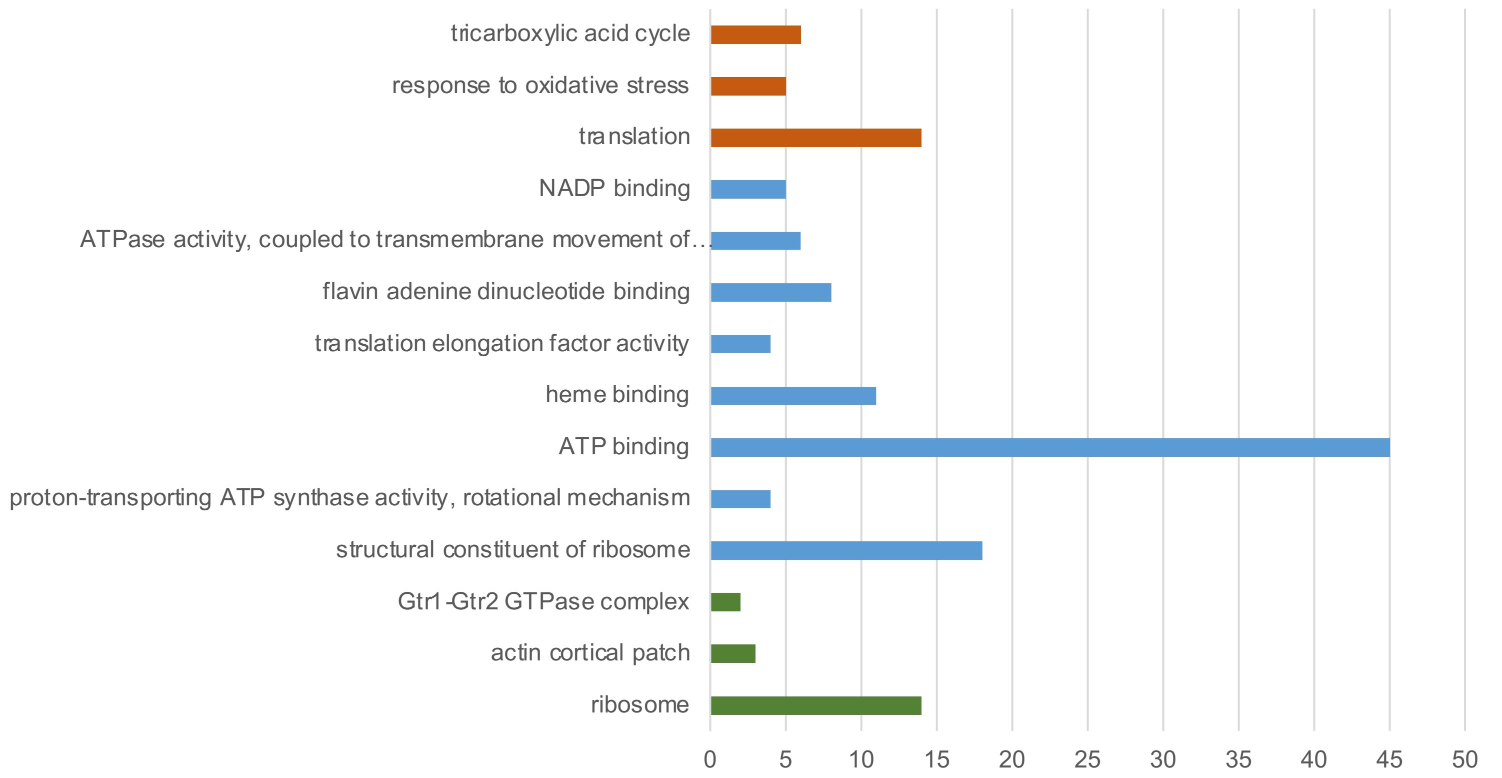

Supplementary Figure S4. Functional analysis of fungal genes expressed in susceptible rootstock (IS). Four hundred forty four genes were clustering based on the gene ontology using DAVID bioinformatics data base (https://david.ncifcrf.gov/home.jsp). The numbers of genes in the functional group of biological process (red), molecular function (blue) and cellular component (green) were displayed.
